# Chondrosarcoma From Floating and Nonfloating Ribs Presenting as a Floating Abdominal Tumor: A Case Report on a Rare Medical Condition

**DOI:** 10.7759/cureus.66091

**Published:** 2024-08-03

**Authors:** Shruthi Bikkumalla, Bhushan Jajoo, Suresh R Chandak, Srinivasa Reddy, Akansha Hatewar

**Affiliations:** 1 Department of General Surgery, Jawaharlal Nehru Medical College, Datta Meghe Institute of Higher Education and Research, Wardha, IND; 2 Department of Surgical Oncology, Jawaharlal Nehru Medical College, Datta Meghe Institute of Higher Education and Research, Wardha, IND

**Keywords:** chest wall tumors, abdominal wall tumors, chest swelling, endosteal scalloping, chondrosarcoma

## Abstract

Chondrosarcoma is a soft tissue tumor that develops in cartilage cells. It can exhibit an aggressive growth tendency when compared to the chondrosarcomas developing in other regions of the body. Clinical presentation of these tumors can also vary depending on the site of presentation. We aim to present the case of a 69-year-old male with a swelling in the chest extending into the abdomen. It is a rare condition that is treated surgically by wide local excision of the tumor.

## Introduction

Chondrosarcoma is a type of soft tissue tumor that develops in cartilage cells. Chondrosarcomas are rare neoplasms and constitute 20%-30% of all malignant bone tumors, with an incidence of one in every 200,000 individuals. Most patients were diagnosed at >40 years of age [1]. The most common bones affected are the sternum, ribs, scapula, costochondral junctions, and pelvis. Variable presentations can often delay the diagnosis, which can, in turn, result in unfavorable outcomes. Chondrosarcoma in the chest wall is almost 25% of all the neoplasms of bone origin in the chest wall [2]. Contrast-enhanced computed tomography (CECT) is recommended as the gold standard diagnostic test and is crucial for decision-making in surgical management [3]. Cortical breach and endosteal scalloping are evident on computed tomography. Though surgical management is the preferred modality, multiapproach treatment incorporating radiation therapy and chemotherapy is often employed to improve local control and overall survival [4].

## Case presentation

**Figure 1:**
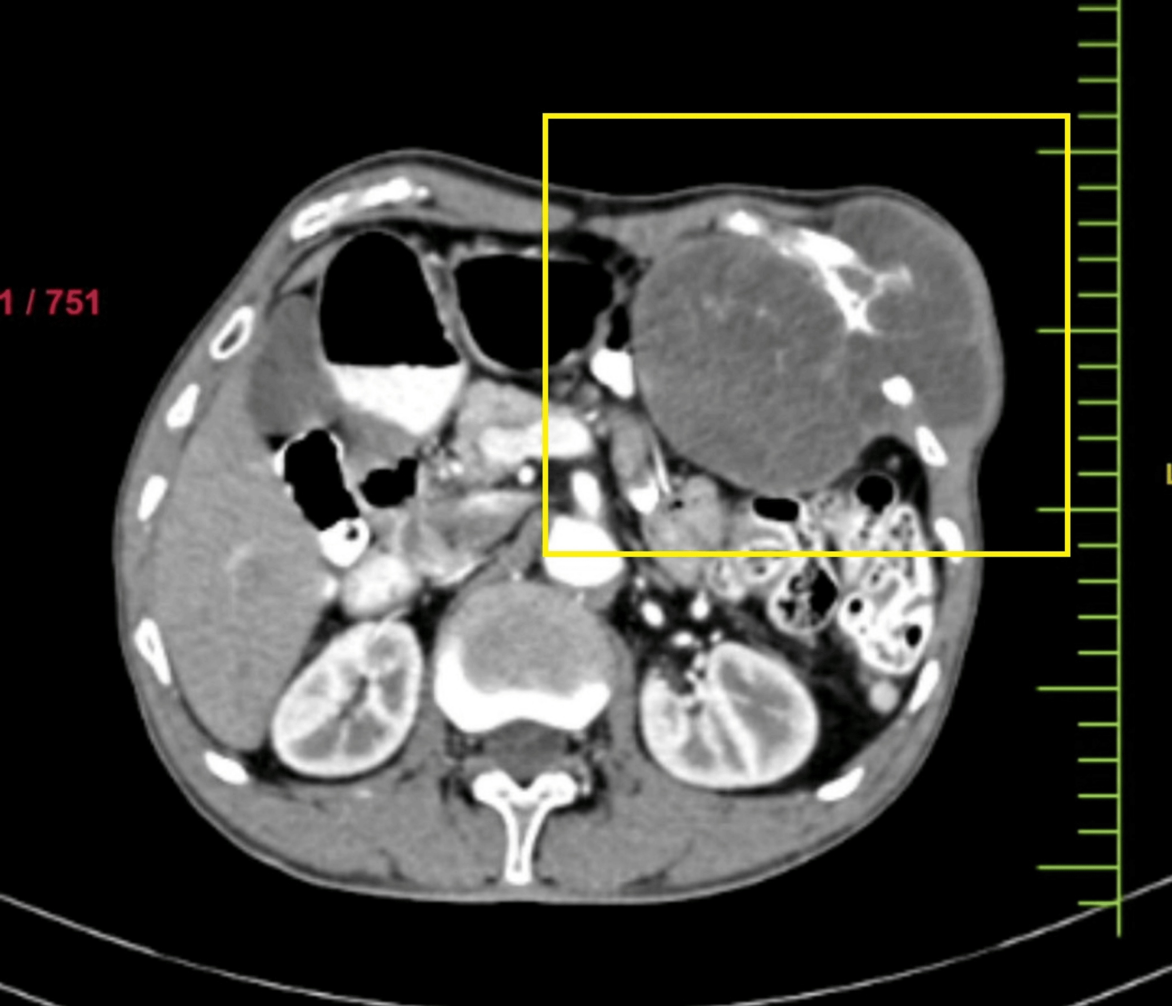
Computerized tomography image showing a lesion intra-abdominally and extra-abdominally involving ribs

A 69-year-old male presented with a complaint of swelling over the left anterior chest wall in the past three years. This was found to be associated with dragging pain, which gradually progressed in size over several months. Physical examination revealed painless, hard, nonmobile swelling with an approximate dimension of 10 x 8 cm in the upper abdomen extending from the left ninth rib to the left hypochondriac region and is adherent to underlying structures with no skin involvement. The biopsy report was suggestive of chondrosarcoma. CECT of the abdomen showed a well-defined lobulated lesion around the lateral aspect of the left ninth rib with significant soft tissue expansion into the left hypochondrium, displacing the stomach, adjacent bowel loops, and the body of the pancreas inferiorly (Figure 1).

**Figure 2:**
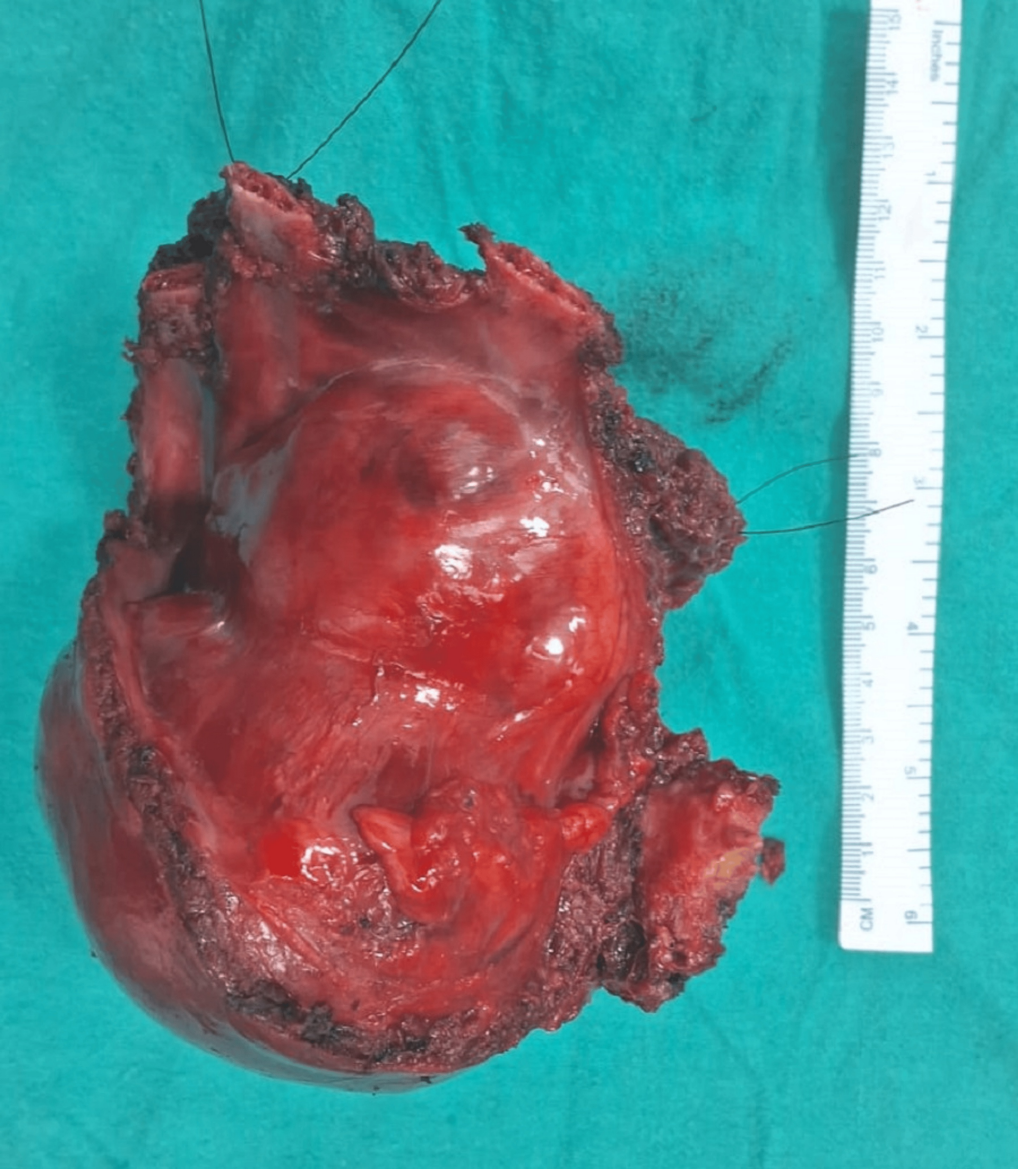
Resected specimen of tumor

**Figure 3:**
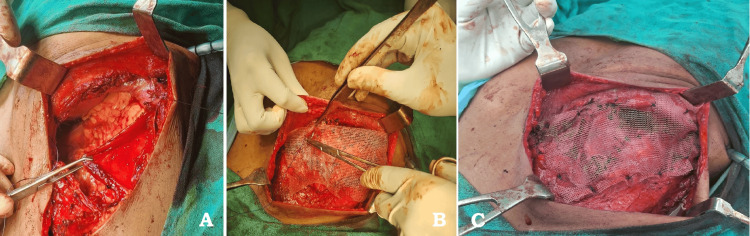
(A) Before mesh placement. (B) Mesh placement. (C) Mesh fixation

The patient was planned for wide local excision of the chest and the intra-abdominal tumor with reconstruction. Intraoperative findings showed that the tumor involving the left 9th, 10th, and 11th ribs near the costochondral junction extending intra-abdominally was excised adequately with a 3-cm margin on the ribs and intra-abdominally from the adherent portion on the diaphragm, thereby exposing the pleural space superiorly and liver laterally. Pleural space has been closed with the remaining diaphragm. There was a lower ribcage defect in the lateral part of the body, which required repair, which was done by Prolene mesh in this patient. The abdominal cavity was covered with peritoneum, and over the peritoneum, Prolene mesh was sutured to intercostal muscles and the remaining part of the diaphragm (Figures 2, 3).

**Figure 4:**
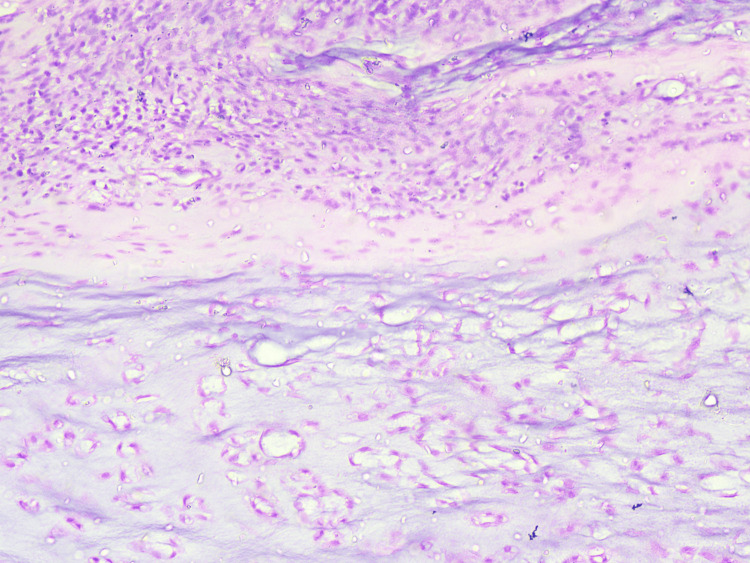
10× resolution of H&E staining of the specimen, which was suggestive of chondrosarcoma H&E: hematoxylin and eosin

**Figure 5:**
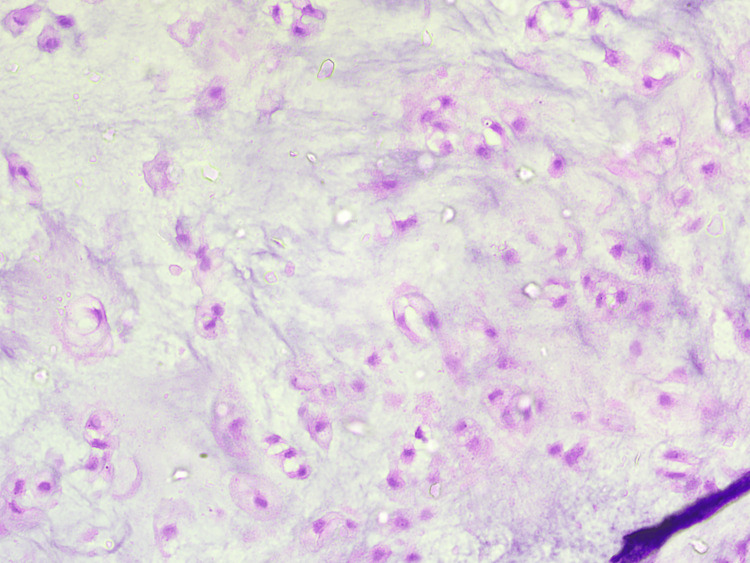
40× magnification of H&E-stained slide of the excised specimen H&E: hematoxylin and eosin

The excised specimen sent for histopathological analysis showed well-differentiated chondrosarcoma of the bone, with all margins found negative for infiltration by malignant epithelial cells (Figures 4, 5).

The patient was doing well at the three-month follow-up. The follow-up was planned three monthly for the first two years, followed by six monthly visits for the next two years.

## Discussion

Though the incidence of chondrosarcoma has been reported low, it is the second most common type of bone tumor with a common clinical presentation of regional dull aching type of pain. It is characterized by its slow growth, commonly noted in individuals of >40 years of age with a slight male predominance [3,5]. Chondrosarcoma may originate from the sternum or the costochondral junction [3], but in this case, it originated from the ribs. In cases of primary chondrosarcoma, there is no defined etiology compared to the incidence of secondary chondrosarcoma, which is known to originate from preexisting enchondromas or osteochondromas [6]. The bones most frequently impacted are the sternum, costochondral junctions, scapula, and ribs. Bones undergoing endochondral ossification are reported to have chondrosarcomas, which commonly originate as de novo lesions or from preexisting benign tumors of cartilaginous origin [7]. Differential diagnosis of sarcoma includes liposarcoma and leiomyosarcoma. Liposarcomas are uncommon mesenchymal neoplasms involving deep soft tissues such as the esophagus, retroperitoneum, and popliteal fossa. For high-grade lesions, wide and deep surgical excision is the mainstay treatment with or without adjuvant radiation and/or chemotherapy. Leiomyosarcoma is soft tissue sarcoma, with an incidence of 1%-4% of the total tumors of the chest wall, with surgery as the primary treatment attributed to its radiation and chemotherapy resistance [8,9].

Tumor aggressiveness and disease prognostics are linked with histopathological grade categorization of the tumor, which has been divided into grades I-IV, which are well differentiated, moderately differentiated, poorly differentiated, and undifferentiated, respectively. Lesion histopathology is commonly studied by hematoxylin and eosin staining, which studies cellular organization, matrix proteins, and cellular divisional stages [10-12]. Standard management protocol recommendations include complete excision of the tumor, which might or might not be associated with adjuvant therapy such as radiotherapy and chemotherapy. The basic treatment of chondrosarcomas is surgical management, and the patient most often requires a surgical resection. Depending on the histopathological report, the patient can be planned for adjuvant chemotherapy. Complete surgical excision with a wide local margin was performed, as conventional chondrosarcomas are not reported to be responsive to standard doses of chemotherapy and radiation [12,13]. Doxorubicin-based adjuvant therapy has been studied in mesenchymal tumors of grades II and III. However, the published literature is based on the results obtained from small and nonrandomized studies [5,13]. Physical examination and radiological imaging are recommended for postoperative surveillance twice a year until five years, followed by an annual screening until 10 years [13,14]. In conclusion, early diagnosis can improve outcomes of better survival and lower recurrence rates.

## Conclusions

Chondrosarcoma including the chest and abdominal wall is rare, usually associated with soft tissue masses. It is a diagnostic challenge and more cases need to be reported which can create an alert and be helpful in early diagnosis and facilitate its clinical management. Its management includes wide local resection of tumor with reconstruction to reduce recurrence and increase disease free survival rates.
